# Burden of malaria among adult patients attending general medical outpatient department and HIV care and treatment clinics in Oromia, Ethiopia: a comparative cross-sectional study

**DOI:** 10.1186/s12936-015-1029-0

**Published:** 2015-12-15

**Authors:** Guda Alemayehu, Zenebe Melaku, Tesfay Abreha, Bereket Alemayehu, Samuel Girma, Yehualashet Tadesse, Tsigereda Gadisa, Sileshi Lulseged, Taye Tolera Balcha, David Hoos, Hiwot Teka, Richard Reithinger

**Affiliations:** U.S. Agency for International Development (USAID), Addis Ababa, Ethiopia; Columbia University ICAP, Addis Ababa, Ethiopia; Columbia University ICAP, New York, USA; Oromia Regional Health Bureau and Federal Ministry of Health Addis Ababa, Addis Ababa, Ethiopia; RTI International, Washington D.C., USA

**Keywords:** Comparative cross-sectional study, Ethiopia, Oromia, HIV, Malaria, Prevalence

## Abstract

**Background:**

Malaria and HIV/AIDS constitute major public health problems in Ethiopia, but the burden associated with malaria-HIV co-infection has not been well documented. In this study, the burden of malaria among HIV positive and HIV negative adult outpatients attending health facilities in Oromia National Regional State, Ethiopia was investigated.

**Methods:**

A comparative cross-sectional study among HIV-positive patients having routine follow-up visits at HIV care and treatment clinics and HIV-seronegative patients attending the general medical outpatient departments in 12 health facilities during the peak malaria transmission season was conducted from September to November, 2011. A total of 3638 patients (1819 from each group) were enrolled in the study. Provider initiated testing and counseling of HIV was performed for 1831 medical outpatients out of whom 1819 were negative and enrolled into the study. Malaria blood microscopy and hemoglobin testing were performed for all 3638 patients. Data was analyzed using descriptive statistics, Chi square test and multivariate logistic regression.

**Results:**

Of the 3638 patients enrolled in the study, malaria parasitaemia was detected in 156 (4.3 %); malaria parasitaemia prevalence was 0.7 % (13/1819) among HIV-seropositive patients and 7.9 % (143/1819) among HIV-seronegative patients. Among HIV-seropositive individuals 65.4 % slept under a mosquito bed net the night before data collection, compared to 59.4 % of HIV-seronegative individuals. A significantly higher proportion of HIV-seropositive malaria-negative patients were on co-trimoxazole (CTX) prophylaxis as compared to HIV-malaria co-infected patients: 82 % (1481/1806) *versus* 46 % (6/13) (P = 0.001). HIV and malaria co-infected patients were less likely to have the classical symptoms of malaria (fever, chills and headache) compared to the HIV-seronegative and malaria positive counterparts. Multivariate logistic regression showed that HIV-seropositive patients who come for routine follow up were less likely to be infected by malaria (OR = 0.23, 95 % CI = 0.09–0.74).

**Conclusion:**

The study documented lower malaria prevalence among the HIV-seropositive attendants who come for routine follow up. Clinical symptoms of malaria were more pronounced among HIV-seronegative than HIV-seropositive patients. This study also re-affirmed the importance of co-trimoxazole in preventing malaria symptoms and parasitaemia among HIV- positive patients.

## Background

Malaria and HIV are major global public health problems, with a particular impact in sub-Saharan Africa. Malaria and HIV share extensive epidemiological overlap, co-infecting large numbers of people in many African countries. Indeed, it has been emphasized that any form of interaction between the two diseases will have a profound implication for treatment, care and prevention programs targeting both diseases [1].

Three-quarters of Ethiopia’s land mass is considered endemic for malaria, with most cases due to *Plasmodium falciparum* and *Plasmodium vivax* being the dominant species. Malaria transmission is seasonal and unstable, the peak transmission season being from September to December. In 2011 the Ethiopian Federal Ministry of Health (FMOH) reported that malaria accounted for up to 17 % of outpatient consultations and 8 % of health facility admissions in the country; 3,898,319 cases were reported [2]. According to the Ethiopian Demographic and Health Survey (EDHS) 2011, the adult HIV prevalence was 1.5 % (1.9 % in women and 1 % in men), with prevalence being 4.2 and 0.6 % in urban and rural areas, respectively [3].

Although malaria and HIV are major causes of morbidity and mortality in the country, the burden of malaria-HIV co-infection in Ethiopia is not well documented. A cross-sectional study conducted in the Hadiya zone, southern Ethiopia, found no difference in the prevalence of HIV among *P. falciparum* positive study participants and the general population [4]. Available evidence on the biological and operational interaction of malaria and HIV is scarce making it difficult to advocate for support in integrated malaria/HIV programming. Additional information is required to determine the burden of malaria among HIV positive persons in order to design appropriate strategies for malaria prevention and control in this population group. The objective of this study was to determine –in an operational programme setting—the burden of malaria infection among HIV-positive and HIV-negative adult population groups in Oromia Regional State, Ethiopia.

## Methods

### Study setting

**Fig. 1:**
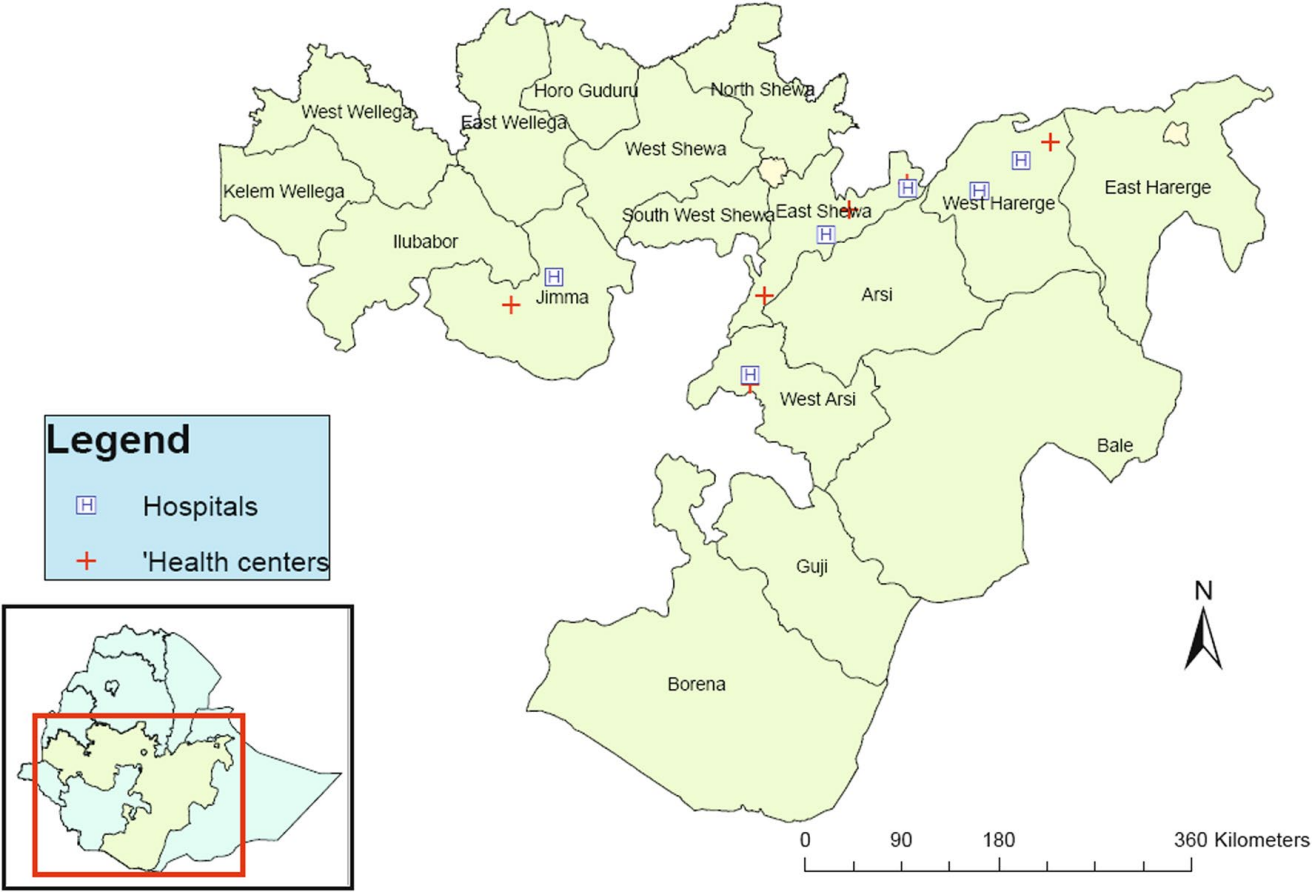
Location of study sites in Oromia National Regional state, Ethiopia

A cross-sectional study was conducted among (1) HIV-negative patients visiting medical outpatient departments (OPD), and (2) HIV-positive patients attending HIV clinics for routine follow-up at 12 health facilities in Oromia from September to November, 2011. Household and school malaria surveys conducted in Oromia reported a parasite prevalence ranging from 0.5 to 10.3 % [5, 6]; both *P. falciparum* and *P. vivax* are endemic in the study sites and the study was conducted during the peak malaria transmission season. HIV prevalence (adjusted for rural/urban) in Oromia was 1.5–1.7 % according HIV sentinel surveillance in 2009 [7] and the EDHS 2011 [3]. Six hospitals (i.e. Limugenet, Shashemene, Metehara, Chiro, Gelemso, and Wonji) and six nearby health centres (i.e. Welenchiti, Shashemene, Metehara, Batu, Hirna, and Agaro) (Fig. 1) were selected for the study based on accessibility, patient load, availability of chronic HIV care and treatment services, and malaria endemicity within their respective catchment areas.

### Sampling method, patient enrollment, clinical and laboratory analysis

Sample size was calculated to compare malaria prevalence in the two patient groups with differing HIV status. Malaria prevalence overall was estimated at 2.5 % based on administrative reports from similar settings, and malaria prevalence among HIV positive individuals was assumed to be 1.7 times the prevalence for HIV negative individuals [8]. Assuming a non-response rate of 2, 80 % power and 5 % significance level, it was estimated that 1818 patients would have to be recruited in each study arm. This sample was proportionally distributed among the twelve study sites based on average quarterly HIV clinic patient load.

**Fig. 2:**
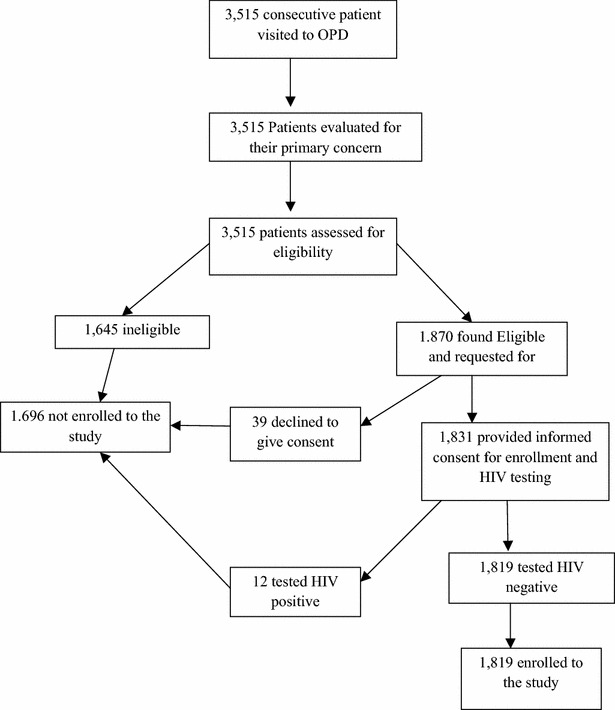
Enrollment of study participants at OPD

**Fig. 3:**
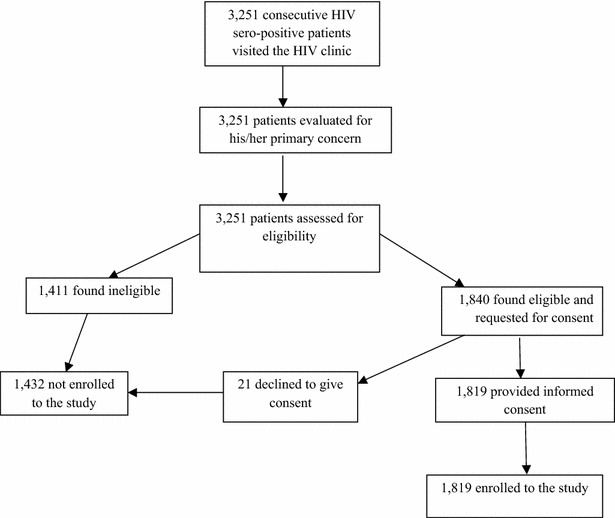
Enrollment of study participants at HIV clinic

Study patients were enrolled from OPDs and the HIV clinics of health facilities. All visiting patients were assessed by health care providers for eligibility and enrollment using inclusion and exclusion criteria set for the study; written consent was obtained from all enrolled patients. Inclusion criteria were patients attending OPDs and HIV clinics, aged ≥18 years, residence in the surrounding malarious villages, patients willing to give consent, patients with no mental illness or altered consciousness, and—in the for OPD patients—willingness to undergo an HIV test. Patients identified at OPD as HIV-positive were excluded from the study population and referred to HIV clinics for routine care (Figs. 2, 3).

Consented patients were interviewed and examined by the study clinician. Physical examination was done for all patients including vital signs, weight and height measurements, and examination of the conjunctiva for pallor. A single finger prick was performed by the laboratory technician and used to prepare one thick and one thin blood film. The slides were stained using 10 % Giemsa for ten minutes and air-dried before examination. Smears were labelled as negative after observing 100 fields at 1000 magnification. When positive for malaria parasites, species and parasite density against 200 leukocytes (assuming an average leukocyte count of 8000 per µl) were determined. Each slide was re-read a second time by an experienced microscopist, blinded to initial microscopy result. A third blinded reading was conducted on all slides with discrepant in first and second slide readings: presence or absence of parasites or difference in species. The third reading was considered final.

HIV testing for patients seen at OPDs was conducted using the national algorithm, which includes three serological tests: KHB (KHB Shanghai Kehua Bio-engineering Co. Ltd, China) as a screening test, STATPAK (Chembio Diagnostics System Inc., USA) as a confirmatory test, and—in case of discordance—UNIGOLD (Trinity Ciotech, USA) as a tie breaker. HemoCue Hb 301 (HemoCue, Angelholm, Sweden) was used to determine the level of hemoglobin in all patients.

### Data management and statistical analysis

A paper data collection instrument was developed, piloted and modified before use in the study. The instrument had three parts: (a) general information about the participants including socio-demographic variables and use of long-lasting insecticidal nets (LLINs); (b) clinical information (e.g. list of anti-malarial drugs used 28 days prior to data collection), laboratory data (e.g. level of haemoglobin, blood film result, parasite species and density) and the treatment given to the patient for a specific problem identified on the same date of diagnosis; and (c) detail of the medications taken by the HIV-positive patients and their CD4 count results.

Data was double entered using SPSS Statistics 17.0 (SPSS Inc., Chicago, USA). Any discrepancy between the two data sets was corrected by referring to the original paper form. The final single data set was cleaned and analyzed using STATA 11.0 (Statacorp, College Station, USA) and SPSS Statistics 17.0 (SPSS Inc., Chicago, USA). Data were organized and summarized using descriptive statistics, Chi square tests, and multivariate logistic regression; P values of <0.05 were considered statistically significant.

### Ethical considerations

Administrative clearance was obtained from all selected study sites and the Oromia Regional Health Bureau. Ethical clearance was obtained from the Institutional Review Boards of Columbia University (ethics number: AAAI2602) and the Ethiopian Public Health Association (ethics number: IORG0004760). All study participants were informed about the objective of the study and written consent was obtained before patient enrollment.

## Results

### Socio-demographic characteristics

A total of 3638 patients were enrolled into the study: half were HIV-negative and half were HIV-positive; equal numbers of HIV-positive and HIV-negative patients were enrolled from most of the facilities. The socio-demographics of the study population are given in Table 1: of study participants, 2006 (55.2 %) were female, 2131 (58.7 %) were married, 748 (20.6 %) were daily laborers, and 2179 (59.9 %) had an income of ≤$30 per month; median age was 33 years (range: 18–99). The income of the poorest quintile accounted for 1 % while it is 55 % for the richest quintile. Malaria prevalence was 6.9 % in the poorest versus 3.3 % in the richest quintile. The proportion of malaria parasitaemia positive patients was higher in the poorest than the richest quintiles (32 vs 15 %, P = 0.18) and proportion of mosquito net availability is lower in the poorest than the richest quintile (15 vs 24 %, P < 0.0001).

**Table 1:** Socio-demographic characteristics of study participants

Socio-demographic variables	No. of HIV negative n (%)	No. of HIV positive n (%)	Total n (%)
Age (in years)			
≤20	303 (16.7)	49 (2.7)	352 (9.7)
21–30	642 (35.3)	664 (36.5)	1306 (35.9)
31-40	391 (21.5)	658 (36.2)	1049 (28.8)
41–50	277 (15.2)	292 (16.5)	569 (15.6)
51–60	141 (7.8)	108 (5.9)	249 (6.8)
61–70	41 (2.3)	39 (2.1)	80 (2.2)
≥71	24 (1.3)	9 (0.5)	33 (0.9)
Sex			
Male	974 (53.6)	657 (36.1)	1631 (44.8)
Female	845 (46.5)	1161 (63.9)	2006 (55.2)
Occupation			
Farmer	491 (27.0)	195 (10.7)	686 (18.9)
Business	85 (4.7)	193 (10.6)	278 (7.6)
Student	213 (11.7)	22 (1.2)	235 (6.5)
Civil servant	406 (22.3)	180 (9.9)	586 (16.1)
Daily laborer	146 (8.0)	602 (33.1)	748 (20.6)
Others	478 (26.3)	626 (34.4)	1104 (30.4)
Marital status			
Married	1218 (67.1)	913 (50.3)	2131 (58.7)
Single	446 (24.6)	176 (9.7)	622 (17.1)
Divorced	56 (3.1)	322 (17.7)	378 (10.4)
Widowed	94 (5.2)	406 (22.3)	500 (13.8)
Income (US $ per month)			
≤30	839 (46.1)	1340 (73.8)	2179 (59.9)
>30	980 (53.9)	479 (26.33)	1459 (40.1)

### Clinical and parasitological findings

Out of the total study participants 156 (4.3 %) had malaria parasitaemia, with *P. falciparum* and *P. vivax* representing 48.1 and 51.9 % of infections respectively. The median of the parasite density was 3060 parasites/µL (range 128–91,555). In 60.9 % of the parasitaemic slides, the parasite load was below 5000 parasites/µL (Table 2).

Of the study patients, 1473 (920 HIV negative and 553 HIV positive) had reported subjective fever. Taking a temperature of 37.5 °C as a cut-off point for febrile status, 15.5 % (283/1816) of the HIV negative patients and 8 % (148/1813) of the HIV positive patients were febrile.

**Table 2:** Malaria species and parasite density among study participants

Category	HIV negative (%) (n = 143)	HIV positive (%) (n = 13)	Total (%) (n = 156)
Malaria parasite species			
*P. falciparum*	69 (48.3)	6 (46.2)	75 (48.1)
*P. vivax*	74 (51.8)	7 (53.9)	81 (51.9)
Parasite density			
≤5000	90 (62.9)	5 (38.5)	95 (60.9)
5001–10,000	24 (16.8)	6 (46.2)	30 (19.2)
10,001–15,000	16 (11.2)	0 (0.0)	16 (10.3)
15,001–20,000	6 (4.2)	0 (0.0)	6 (3.9)
≥20,001	7 (4.9)	2 (15.4)	9 (5.8)

The mean haemoglobin value for the 156 malaria positive patients was 13.93 mg/dl, while it was 14.06 for the rest. Again, the mean hemoglobin value of the 283 OPD malaria febrile patients was 14.2, it was 14.56 for the remaining individuals. Moreover, mean haemoglobin for HIV malaria co-infected was 12.72, where as for HIV mono-infected individuals it was 13.62 mg/dl.

Malaria parasitaemia prevalence was 7.9 % (143/1819) in HIV-negative patients. The geometric mean of the parasite count in this group was 2933 (95 % CI 2371–3626), where as parasitaemia prevalence was 0.7 % (13/1819) in the HIV-positive patients. The geometric mean of the parasite count in this group was 4931 (95 % CI 2028–12,004). The parasite load in 53/143 (37.1 %) of the malaria parasite positive samples from HIV-negative patients was >5000 parasites/µL, however, parasite load in 8/13 (61.5 %) of the HIV-positive patients was >5000 parasites/µL.

The majority of the HIV-positive patients 1419 (78 %) were on antiretroviral treatment (ART), 52.3 % of them for less than 2 years. Being on ART was not associated with protection from malaria infection among HIV positive individuals. Similarly, 1473 (81 %) of them were on co-trimoxazole prophylactic therapy (CPT), 54 % of them for less than 2 years. A significant difference in CPT use was noted among malaria parasitaemia positive (6/13, 46 %) and malaria parasitaemia negative 1481/1806, (82 %) HIV positive patients (P = 0.001) as shown in Table 3.

**Table 3:** Characteristics of HIV positive malaria co-infected patients compared to HIV positive patients without malaria infection

Patient characteristics	Blood film positive (n = 13)	Blood film negative (n = 1806)	P value
Percent male	46 %	36 %	0.45
Mean age	37.76	36.01	0.54
% With mosquito net at home	54 %	54 %	0.98
% Use of mosquito net	31 %	35 %	0.73
% On CPT	46 %	82 %	0.001
% On ART	69 %	78 %	0.44
Median (IQR) cd4 count (cells/mm^3^)	493 (297–551)	356 (231–524)	0.08
Mean haemoglobin level	12.72	13.62	0.10

*IQR* inter-quartile range

### Malaria patients

**Fig. 4:**
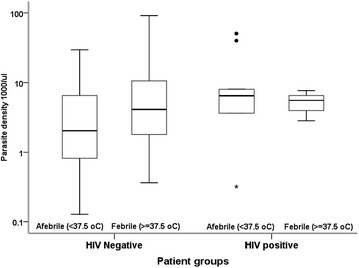
*Box* and *whisker* plots of log10 parasite densities (parasites × 10^3^/µL) comparing HIV negative and HIV positive patients by afebrile and febrile strata. *Boxes* represent the interquartile range, *whiskers* are a maximum of 1.5 times the interquartile range, outliers are shown as *circles* and *stars*

HIV-positive, malaria co-infected patients were less likely to have fever [OR = 0.24 (95 % CI 0.06–0.95), P = 0.04] or a headache [OR = 0.15 (95 % CI 0.04–0.61)]; HIV-positive patients had lower mean hemoglobin levels [mean difference = 1.32 (95 % CI 0.2–2.45), P = 0.02] compared to HIV-negative malaria-infected patients (Table 4). Overall, there was no significant difference between the parasite densities between the two patient groups, including when adjusted by fever status (Fig. 4).

**Table 4:** Clinical characteristics of blood film positive study participants

Patient characteristics	Malaria positive		*P* value
	HIV negative (n = 143)	HIV positive (n = 13)	
% With reported fever	83 %	54 %	*0.04*
% With chills	78 %	54 %	0.14
% With headache	88 %	54 %	*0.002*
Mean temperature (°C)	36.8	36.4	0.24
Febrile (≥37.5 °C)	41.5 %	23.1 %	0.19
Systolic blood pressure (mmHg)	106.6	106.9	0.95
Diastolic blood pressure (mmHg)	72.1	74.6	0.45
Mean haemoglobin level (gm/dl)	14.0	12.7	*0.02*

### Risk factors

Among all the study participants, 2155 (59.2 %) reported that they owned LLINs, and ownership was higher in the HIV-negative group than the HIV positive study group 1167 (64 %) vs 988 (54 %), χ^2^ = 36.47, P < 0.0001. Of the patients who reported LLINs ownership, 1335 (62 %) reported that they had slept under it the night before the survey, 65.4 % in the HIV-positive and 59.4 % in the HIV-negative individuals (P = 0.004). There was no significant difference in LLIN use between malaria-HIV co-infected and HIV infected patients (31 vs 35 %, P = 0.73). Potential confounders including age, sex, occupation, income, family size, ownership and use of an LLIN, use of anti-malarial drugs, [i.e. ACT (Coartem^®^), chloroquine, doxycycline, sulfadoxine-pyrimethamine, quinine], ART and CPT were assessed. The crude odds ratio (OR) for the association between malaria parasitaemia and patient’s HIV status was 0.08 (95 % CI = 0.04–0.14). Difference of the adjusted OR was considered if the crude OR was found beyond ±20 %. Only two variables (CPT and ART) were labelled as confounders. Based on the set criteria, the crude and adjusted ORs for the two variables (CPT and ART) differed. The crude and adjusted ORs including CPT and ART were 0.08 (95 % CI = 0.04–0.14) and 0.25 (95 % CI = 0.11–0.53), respectively. Finally, a multivariate logistic regression analysis including the confounders in the model resulted in an OR of 0.23 (95 % CI = 0.09–0.74) as shown in Tables 5 and 6.

**Table 5:** Patient characteristics associated in univariate analysis with blood film result among the study participants

Variables	OR	95 % CI
Age		
≤20	1	Reference
21–30	0.33	0.22–0.50
31–40	0.19	0.11–0–31
41–50	0.21	0.12–0.39
51–60	0.34	0.17–0.68
61–70	0.19	0.04–0.81
≥71	0.74	0.21–2.52
Sex		
Male	1	Reference
Female	0.45	0.32–0.63
Occupation		
Farmer	1	Reference
Business	0.2	0.07–0.57
Student	1.96	1.20–3.21
Civil servant	0.52	0.30–0.88
Daily labourer	0.34	0.20–0–60
Others	0.49	0.32–0.77
Income		
≤$30.00/month	1	Reference
>$30.00/month	1.07	0.77–1.48
Family size		
1–5	1	Reference
6–10	1.25	0.88–1.78
11+	0.71	0.17–2.94
TMP/SMX		
No	1	Reference
Yes	0.05	0.02–0.12
Sleep under net		
No	1	Reference
Yes	0.67	0.5–0.95
Sulfadoxine-pyrimethamine		
No	1	Reference
Yes	0.00	–
Quinine		
No	1	Reference
Yes	0.00	–
Coartem		
No	1	Reference
Yes	4.90	2.24–10.71
Chloroquine		
No	1	Reference
Yes	4.04	1.78–9.19
Doxycycline		
No	1	Reference
Yes	0.77	0.10–5.68
ART		
No	1	Reference
Yes	0.09	0.04–0.18

*TMP/SMX* trimethoprim/sulfamethoxazole, *ART* anti-retroviral therapy

**Table 6:** Potential confounders which may distort the association between malaria parasitaemia and HIV status of patients

Variable	Adjusted odds ratio	
	OR	95 % CI
Age	0.09	0.05–0.17
Sex	0.09	0.05–0.16
Occupation	0.1	0.05–0.18
Income	0.09	0.05–0.15
Family size	0.08	0.05–0.15
TMP/SMX	0.25	0.11–0.53
Net use	0.08	0.04–0.14
Coartem	0.09	0.05–0.16
Chloroquine	0.08	0.04–0.15
Doxycycline	0.08	0.04–0.14
Sulfadoxine-pyrimethamine	0.08	0.04–0.14
Quinine	0.08	0.04–0.14
ART	0.11	0.04–0.32

## Discussion

Until 1998, there was “no convincing evidence for an interaction between malaria and HIV” [9]. Accumulated evidences revealed that HIV positive people in areas of malaria transmission have more frequent episodes of symptomatic malaria and higher parasitaemia than those without HIV [10, 11]. By 2006, it was estimated that the interaction of malaria and HIV in one Kenyan district alone had caused 980,000 excess malaria episodes and 8500 excess HIV infections since HIV’s emergence in the 1980s [12]. Malaria is more common and severe in adults with HIV, pregnant women, and children. The study on interaction of malaria and HIV in Africa has shown that HIV viral load is greater in women with placental malaria, and infants born to women with both HIV and placental malaria are of greater risk of premature delivery, low birth weight, or death compared with infants born to women with HIV infection alone. The study also showed malaria causes increased HIV viral load in blood and breast milk and reduced CD4 cell count [13]. Malaria treatment is less effective in non-pregnant adults with HIV [14].

The current study demonstrated that—in an operational setting with scaled-up HIV/AIDS services and a setting of seasonal malaria transmission—HIV-positive patients attending HIV specialty clinics were less likely to have malaria parasitaemia compared to HIV-negative patients attending general OPD clinics during peak malaria transmission season (OR = 0.23; 95 % CI = 0.09–0.74). In this study, the malaria prevalence in HIV-positive patients was similar (i.e. 0.7 %) to the 0.5 % prevalence among the general population in Oromia as reported from the national Malaria Indicator Survey (MIS) 2011 [6] conducted in the same transmission season. The MIS survey was a cross-sectional study that screened the population within households for malaria parasitaemia regardless of whether or not there were any acute malaria illness symptoms. In this study, many general medical outpatient department patients were self-selected due to the presence of acute symptoms of illness that required immediate relief, whereas the HIV positive patients attending HIV specialty clinic were more likely to be receiving routine medical care follow-up and health maintenance including medication refills.

In a prospective study conducted in 196 patients using rapid antigen diagnostic tests in Nigeria, the prevalence rate of *P. falciparum* malaria among HIV sero-negative group was 10.6 % and amongst HIV sero-positives was 18.9 % [15]. In addition, studies conducted elsewhere in higher malaria transmission settings strongly suggested increased risk of malaria in HIV infected individuals [10, 11, 16–18]. According to results on malaria HIV co-infection in eastern sub-Saharan Africa, individuals who lived in areas with high *P. falciparum* parasite rate have about twice the risk of being HIV positive compared with individuals who live in areas with low *P. falciparum* parasite rate [19]. In this study, both *P. falciparum* and *P. vivax* parasites were identified with similar distribution and parasite densities among malaria HIV co-infected and malaria mono-infected patients.

Although, a higher mean parasite density was observed in the febrile as compared to non-febrile patients and among HIV-positive and HIV-negative patients, no statistically significant difference was observed. Although there is a paucity of comparable evidence from the same setting, a study conducted by Cheryl et al. in South Africa demonstrated no significant difference in parasite count in HIV negative and positive individuals [20]. However, Tatfang et al. reported a significant difference in mean parasite count between the two patient groups [21].

This study also showed that HIV and malaria co-infected patients were less likely to have the classical symptoms of malaria (fever, chills and headache) compared to the HIV negative—malaria positive patients. Immunological reasons may have contributed for this finding. HIV patients’ lesser ability to manifest with the typical symptoms of malaria, which heralds a high index of suspicion could have a profound implication in clinical diagnosis. In contrast to the findings of this study, many studies conducted in malaria endemic regions indicated an increased risk of clinical malaria in HIV infected individuals consistent with the level of immune-suppression [11, 18, 22, 23]. This study has noted that the mean hemoglobin level of HIV-malaria co-infected patients was also significantly lower than the malaria mono-infected patients (12.7 vs 14.0 g/dl, P = 0.02). This is similar with the results of a study conducted by Nkuo-Akenji et al. that showed more prevalent anaemia in the malaria-HIV co-infected than in cases mono-infected with either HIV or malaria [24].

Some of the socio-demographic factors that included age, sex, occupation, marital status, and income were not found to be contributing to the difference observed in malaria prevalence among HIV-negative and positive study groups. Though not statistically significant, the proportion of malaria positive patients was higher in the poorest quintiles of the study participants compared to the richest, while the mosquito net availability was significantly lower in the poorest than the richest quintile. Studies conducted so far have suggested that malaria should be regarded as a disease of the poor or a disease of poverty as it was always noted that the concentration of the disease was in poorest continents and countries [25]. Nevertheless, in a Malaria Indicator Survey conducted in Ethiopia in 2007, a 0.6 % of malaria prevalence was found in the poorest quintile as compared to 1.6 % in the richest quintile [26].

The HIV-positive patients compared to the HIV-negative patients were observed to have frequently slept under the mosquito net (P = 0.004). The utilization of mosquito nets among the HIV positive study participants was not found to be protective for malaria infection. This result seems to be different from reports by other studies conducted elsewhere which could be due to the fact that this study might have been limited by the small number of malaria positives encountered among the HIV positive study participants. Although sleeping under mosquito nets was not significantly different among malaria-HIV co-infected and HIV mono-infected patients, the provision of CPT to the HIV positive patients have significantly prevented infection from malaria. This concurs with the findings of a prospective cohort study conducted in Uganda which showed that the provision of CPT, ART, and bed nets was associated with a 95 % fall in the frequency of malaria from 50.8 to 2.1 episodes per 100 person-years in HIV positive adults. The addition of ART to a cohort of HIV positive people already receiving CPT was associated with a 64 % fall in malaria incidence [27].

The presence of sub microscopic infections in low endemic settings like Ethiopia is increasingly getting attention. The study in Ethiopia on sub-microscopic carriage of *P. falciparum* and *P. vivax* in low endemic areas showed that malaria infections were not detected by microscopy despite >5 % parasite prevalence detected by nested poymerase chain reaction (nPCR). In this study all participants were malaria negative for microscopy and RDT. Nested PCR revealed *P. falciparum* mono-infection in 5.2 % (29/555), *P. vivax* mono-infection in 4.3 % (24/555), where as mixed infection was 0.2 % (1/555) [28] . Similarly, in the current study no mixed infection was observed and microscopy was used which is usually insensitive for low-density parasitaemia. Molecular techniques, on the other hand, allow the detection of low-level, sub-microscopy parasitaemia. Another study in Ethiopia has similarly showed that the prevalence of sub-microscopic *P. falciparum* carriage was 19.2 % (77/400) out of RDT and microscopy negative blood samples, while microscopy based prevalence of *P. falciparum* infection was 3.7 % (54/1453) [29].

This study has some limitations. Due to small number of HIV malaria co-infected individuals (n = 13) caution should be exercised in interpretation and further analysis. The HIV-negative study participants were visiting health facilities at OPD to seek medical attention on self-reported acute symptomatic illnesses, while the HIV positive patients came to the HIV clinic for a routine medical follow up. The differences in the malaria prevalence and clinical presentations in both study groups could, to some degree, be attributed to the inherent differences of the reasons for clinic attendance between the two study populations.

## Conclusion

This study was conducted in an operational setting with scaled-up HIV/AIDS services and seasonal malaria transmission. This study showed lower malaria prevalence in HIV-positive than HIV-negative patients. The prevalence of malaria in the HIV-positive population was similar to the general population’s malaria prevalence that was measured in the national MIS 2011. Use of insecticide-treated mosquito nets was reportedly greater in the HIV-positive than the HIV-negative study groups. This study reinforces the importance of CPT in preventing malaria among HIV positive patients.
